# Near-infrared imaging for visualizing the synergistic relationship between autophagy and NFS1 protein during multidrug resistance using an ICT–TICT integrated platform†

**DOI:** 10.1039/d3sc06459j

**Published:** 2024-03-25

**Authors:** Wei Hu, Yifan He, Haixian Ren, Li Chai, Haiyan Li, Jianbin Chen, Chunya Li, Yanying Wang, Tony D. James

**Affiliations:** a Key Laboratory of Catalysis and Energy Materials Chemistry of Ministry of Education, Key Laboratory of Analytical Chemistry of the State Ethnic Affairs Commission, College of Chemistry and Materials Science, South-Central Minzu University Wuhan 430074 China lichychem@mail.scuec.edu.cn wangyychem@mail.scuec.edu.cn; b Department of Chemistry, Xinzhou Normal University Xinzhou Shanxi 034000 China hxren326@163.com; c Department of Chemistry, University of Bath Bath BA27AY UK t.d.james@bath.ac.uk; d School of Chemistry and Chemical Engineering, Qilu University of Technology (Shandong Academy of Sciences) Jinan Shandong 250353 China; e School of Chemistry and Chemical Engineering, Henan Normal University Xinxiang 453007 China

## Abstract

Drug resistance is a major challenge for cancer treatment, and its identification is crucial for medical research. However, since drug resistance is a multi-faceted phenomenon, it is important to simultaneously evaluate multiple target fluctuations. Recently developed fluorescence-based probes that can simultaneously respond to multiple targets offer many advantages for real-time and *in situ* monitoring of cellular metabolism, including ease of operation, rapid reporting, and their non-invasive nature. As such we developed a dual-response platform (Vis-H_2_S) with integrated ICT–TICT to image H_2_S and viscosity in mitochondria, which could simultaneously track fluctuations in cysteine desulfurase (NFS1 protein and H_2_S inducer) and autophagy during chemotherapy-induced multidrug resistance. This platform could monitor multiple endogenous metabolites and the synergistic relationship between autophagy and NFS1 protein during multidrug resistance induced by chemotherapy. The results indicated that chemotherapeutic drugs simultaneously up-regulate the levels of NFS1 protein and autophagy. It was also found that the NFS1 protein was linked with autophagy, which eventually led to multidrug resistance. As such, this platform could serve as an effective tool for the in-depth exploration of drug resistance mechanisms.

## Abstract 2

Drug resistance is a major challenge for cancer treatment, and its identification is crucial for medical research.

## Graphical abstract

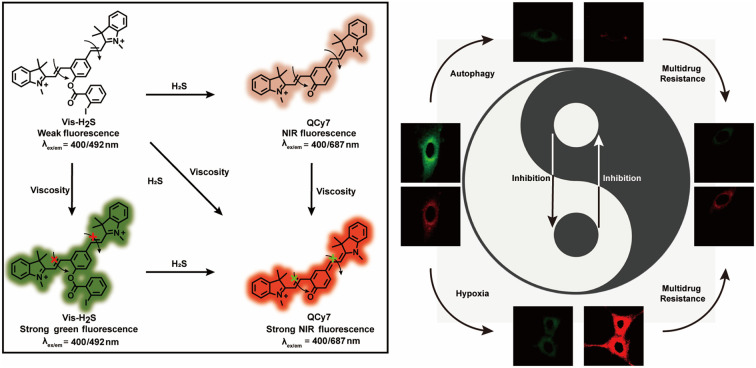

## Introduction

Chemotherapy, the main method for treating malignant tumours, usually produces significant drug resistance during the later stages of treatment.^1^ This reduces therapeutic effects and even results in resistance to alternative drugs that use different mechanisms, which is termed multidrug resistance (MDR).^2^ MDR can interfere with the intake and accumulation of drugs in tumour cells, reducing the ability of anticancer drugs to induce cell death, resulting in drug inactivation and degradation, and the activation of anti-apoptosis and antioxidant defence, as well as DNA repair and replication. These factors are the main causes of the failure of chemotherapy.^3^ Common tumour MDR detection methods include MTT assays, drug sensitivity tests, MDR gene and pathway detection, and high-throughput screening technologies.^4^ However, using these methods makes it impossible to achieve *in situ*, real-time measurements and accurate detection of MDR in tumour cells. The methods are also expensive and time-consuming, which significantly limits their application for the evaluation of MDR mechanisms.

Confocal fluorescence imaging requires simple operation and exhibits high sensitivity and temporal and spatial resolution and can provide *in situ* and real-time analysis.^5^ However, fluorescence-based sensors based on traditional sensing mechanisms have only one recognition site and can only recognize one analyte due to their single mode sensing mechanism.^6^ These are unsuitable for evaluating multiple interconnected biomolecules in complex living systems. To overcome this deficiency, fluorescence-based sensors have emerged that use two or more sensing mechanisms.^7,8^

Chemotherapeutic drugs which increase the amounts of reactive oxygen species (ROS) (such as doxorubicin and cisplatin) can kill tumour cells. However, in addition they induce many factors that can result in MDR, including the activation of autophagy^9^ (caused by excessive ROS, which then prevents cancer cell apoptosis by degrading the ROS) and up-regulating the levels of cysteine desulfurase^10^ (NFS1 protein, which can catalyse cysteine to produce endogenous hydrogen sulfide and consume ROS, which prevents cancer cell apoptosis). However, the synergistic relationship between autophagy and NFS1 protein overexpression *via* ROS-based chemotherapeutic drugs has not yet been reported.

To achieve the *in situ* imaging of the synergistic relationship between autophagy and reductive protein overexpression, a probe with mitochondrial anchoring ability with a dual-response toward viscosity (changes in mitochondrial viscosity can indicate autophagy^11^) and H_2_S (mitochondrial H_2_S fluctuations may indicate the NFS1 protein level^12^) is required. With this research, a cyanine dye QCy7 with mitochondrial anchoring ability was used as the parent fluorophore^13^ and 2-iodobenzoate was used as the specific recognition site for H_2_S (Vis-H_2_S). When Vis-H_2_S reacts with H_2_S, intramolecular charge transfer (ICT) is activated and the fluorescence emission wavelength red-shifts from 492 nm to 687 nm, allowing it to be used to monitor changes in the H_2_S concentration, while the double bond contained in QCy7 can undergo twisted intramolecular charge transfer (TICT), resulting in a significant decrease in the fluorescence intensity, but this can be inhibited by a high-viscosity environment. Therefore, when the viscosity increased from 0.903 cp to 965 cp, the fluorescence intensity at 492 nm increased 110-fold. This enabled the monitoring of environmental viscosity. Using the ICT–TICT dual-response strategy, Vis-H_2_S could simultaneously respond to mitochondrial viscosity and H_2_S fluctuations. Thus, it can be used to explore the synergistic relationship between autophagy and the NFS1 protein.

## Results and discussion

### Design and synthesis of Vis-H_2_S

**Scheme 1:**
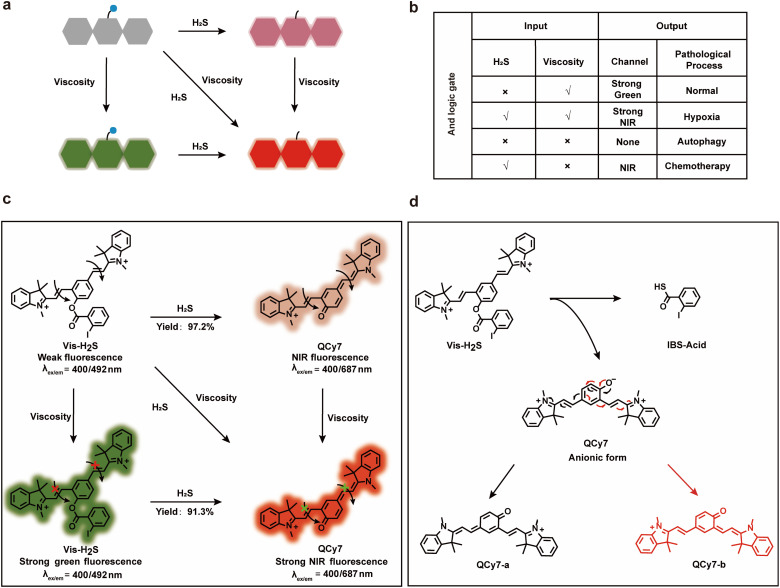
Schematic illustration (a) and “AND” logic gate (b) of the dual-response platform; (c) the response mechanism of Vis-H_2_S toward H_2_S and viscosity; (d) the sensing mechanism of H_2_S using Vis-H_2_S.

As mentioned above, the “AND” logic gate-based “ICT–TICT dual-response mechanism” probe (Vis-H_2_S) requires the coexistence of two analytes (viscosity and H_2_S) and as such can monitor the synergistic relationship between autophagy and NFS1 protein. As shown in Scheme 1a and b, Vis-H_2_S generated NIR fluorophores under the simultaneous activation by two agents, *i.e.*, H_2_S and viscosity. In a low viscosity environment, weak NIR fluorescence of Vis-H_2_S was observed in the presence of H_2_S, while in the presence of high viscosity (autophagy-regulates mitochondrial viscosity) and the absence of H_2_S, Vis-H_2_S emits remarkable green fluorescence.

If, however, both high viscosity and H_2_S are present, it emits strong near-infrared fluorescence. For normal cells, H_2_S is present at a low level, and mitochondria have a high viscosity due to the presence of many proteins (resulting in strong green fluorescence), but H_2_S and mitochondrial viscosity are easily destroyed by hypoxia and autophagy, respectively. Increased cell hypoxia can lead to the overexpression of reducing proteins such as NFS1 protein which results in a strong NIR fluorescence response. On the other hand, autophagy reduces the viscosity of the mitochondria, resulting in minimal fluorescence from either the red or green channel. As such, MDR produced by chemotherapy can lead to a simultaneous increase in autophagy and hypoxia, resulting in only weak NIR fluorescence from the system. The response mechanism is provided in Scheme 1c and d. Vis-H_2_S, with a molecular rotor structure (two symmetric vinyl-coupled indolium moieties), enhanced the probe fluorescence in a high-viscosity environment due to restricted intramolecular rotation inhibiting nonradiative twisted intramolecular charge transfer (TICT^14–16^). The nucleophilic reaction of the ester with HS^−^ releases QCy7 in an anionic form, and the negative charge is delocalized to form quinone QCy7-a or QCy7-b.

Vis-H_2_S was synthesized according to Scheme S1,† and characterization data are provided in Fig. S1–S4.† The reaction mechanism between Vis-H_2_S and H_2_S was verified by measuring the relative molecular mass before and after the reaction using HR-MS. As shown in Fig. S5,† when Vis-H_2_S was reacted with H_2_S, a peak appeared at *m*/*z* = 461.2098, representing the reaction product and the peak at *m*/*z* = 346.1212 (representing the reactant) disappeared. We concurrently employed ^1^H-NMR spectroscopy to conduct a mechanistic analysis of Vis-H_2_S's response to H_2_S. The results are illustrated in Fig. S6,† and exhibit significant chemical shift variations in the methyl hydrogens (H_a_, H_b_, H_c_ and H_d_) on the indole moiety of the probe. Simultaneously, the signals corresponding to the hydrogens from the iodinated benzene disappear, substantiating the validity of the predicted mechanism underlying the response of Vis-H_2_S's to H_2_S. To further investigate the response of Vis-H_2_S and QCy7 to viscosity, a PBS–glycerol solvent system was employed to monitor the spectral response of Vis-H_2_S to viscosity changes (Fig. S7 and S8†). Vis-H_2_S and QCy7 exhibited a nearly 110-fold and 22-fold increase in fluorescence, respectively, in response to the increase in solution viscosity from 0.903 cP to 965 cP. The relative fluorescence intensity of Vis-H_2_S and QCy7 exhibited a linear relationship with viscosity (*R*^2^ = 0.985 for Vis-H_2_S and *R*^2^ = 0.984 for QCy7). The above results confirmed that Vis-H_2_S and QCy7 exhibit excellent responses to viscosity and can be used to detect viscosity changes during *in vivo* and *in vitro* experiments. Additionally, we delved into the polarity responsiveness of QCy7 by examining its behavior in PBS–THF mixtures with different volume fractions (*f*_w_) of PBS, spanning the range from 10% to 100%. As shown in Fig. S9,† there was minimal change in fluorescence intensity, indicating that QCy7 is not sensitive to polarity.

**Fig. 1:**
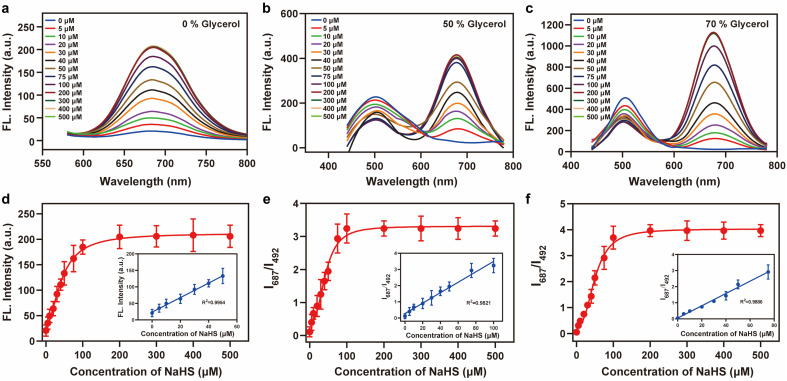
Fluorescence intensity changes of Vis-H_2_S (10 μM) in response to H_2_S and viscosity. (a) 0% glycerol, (b) 50% glycerol, and (c) 70% glycerol; fluorescence titration curves of the probes Vis-H_2_S and H_2_S (0–500 μM); (d)–(f) the fluorescence intensity responses of Vis-H_2_S for (a)–(c) to different concentrations of H_2_S (0–500 μM) at the ratio signal (*I*_687 nm_/*I*_492 nm_), respectively. The inset shows the linear relationship of the Vis-H_2_S response to a low concentration of H_2_S in different glycerol systems. The excitation wavelength was 400 nm, and the test medium was phosphate buffer (10 mM; pH = 7.4).

2-Iodobenzoate was used to regulate the “push–pull” electronic effects (intramolecular charge transfer, ICT) of Vis-H_2_S so that it could specifically respond to changes in H_2_S. We initially examined the absorption and fluorescence spectra of Vis-H_2_S in response to H_2_S. The results revealed that Vis-H_2_S initially exhibited low levels of absorption and fluorescence intensity, but a significant increase was observed upon the addition of H_2_S (Fig. S10a and b†). We also investigated the response of Vis-H_2_S to H_2_S in environments with different viscosities (PBS, pH = 7.4, containing 0%, 50%, and 70% glycerol). The ability of Vis-H_2_S to simultaneously respond to H_2_S and viscosity was also evaluated. As shown in Fig. S10a† and 1, upon increasing the H_2_S concentration in the absence of glycerol, Vis-H_2_S*vs.* H_2_S exhibited a “turn-on” response, with an approximately 20-fold increase in the fluorescence intensity at 687 nm. Upon increasing the glycerol concentration, a ratiometric sign was found with increasing concentrations of H_2_S (Vis-H_2_S exhibited a decreasing intensity at *λ*_em_ = 492 nm and an increasing intensity at *λ*_em_ = 687 nm). Upon increasing the glycerol concentration, *I*_green_ and *I*_red_ both increased significantly. The ratio signal also exhibited a good linear relationship at 50% and 70% glycerol when the H_2_S concentration was lower than 75 μM. From Fig. 1e and f the correlation coefficients were 0.982 and 0.989, respectively, and the limit of detection (LOD) values were calculated to be 45 nM and 50 nM, respectively, according to 3*σ*/*k* (where *σ* is the standard deviation of the blank (*n* = 11) and *k* is the slope for the range of linearity). The above experiments confirmed the dual-response of Vis-H_2_S to viscosity and H_2_S. Kinetic experiments indicated that when using 70% glycerol, the reaction between Vis-H_2_S and H_2_S (100 μM) reached a plateau within 60 min, indicating that Vis-H_2_S could rapidly recognize H_2_S (Fig. S10f†). Eighteen different interferents were selected to evaluate the selectivity of Vis-H_2_S. As shown in Fig. S10g,† these interferents did not change the fluorescence intensity of Vis-H_2_S in either a high-viscosity (glycerol) or low-viscosity (PBS) environment. These results clearly indicate that Vis-H_2_S exhibits minimal interference and can be used for *in situ* imaging of cellular viscosity. Finally, we evaluated the effect of pH on the response of Vis-H_2_S to H_2_S. As shown in Fig. S10c,† for pH over a range from 4–8, there was no obvious change in the fluorescence signal before or after the simultaneous action of Vis-H_2_S with H_2_S and glycerol. In addition, the interference of cell viscosity stimulators (temperature and monensin^17^) on the fluorescence intensity of Vis-H_2_S was investigated. The results in Fig. S10d† show that neither temperature nor monensin interfered with the fluorescence intensity of Vis-H_2_S, which provides an excellent basis for subsequent cell experiments. According to the literature,^18^ acylphenol can serve as a recognition moiety for carboxylesterase-2, which is structurally similar to our recognition domain. To exclude the potential interference of carboxylesterase-2 in this study, we conducted photostability experiments of Vis-H_2_S in response to carboxylesterase-2, as shown in Fig. S10f.† The results indicate that over time, there was no change in fluorescence intensity, suggesting that carboxylesterase-2 has no interference with H_2_S.

With the above results in hand, we evaluated the *in situ* imaging capability of Vis-H_2_S for H_2_S in cells. First, different concentrations of Vis-H_2_S were incubated with HepG2 cells for 24 h. The MTT assay results indicated that the cell viability remained above 80% and the IC_50_ was 63 μM, which indicates that Vis-H_2_S was not cytotoxic and could be used for cell imaging (Fig. S11†). To accurately image autophagy and NFS1 protein simultaneously, the probe needs to exhibit mitochondrial-anchoring ability. According to the literature, as an organelle with a double-membrane structure, the negative charge of the inner mitochondrial membrane enables positively charged molecules to be specifically enriched and anchored in the mitochondria.^19^ Since the indole group of the Vis-H_2_S probe is positively charged, the intracellular distribution should be targeted to the mitochondria. To this end, Vis-H_2_S was mixed with a commercial mitochondrial localization dye, 100 nM Mito-Tracker Red, a commercial lysosome localization dye, 100 nM Lyso-Tracker Red, and a commercial endoplasmic reticulum localization dye, 100 nM ER-Tracker Red. These were then co-incubated with HepG2 cells for 30 min and imaged using confocal microscopy. As shown in Fig. S12†, Vis-H_2_S was mainly distributed in the mitochondria (Pearson colocalization coefficient was 0.94), while the Pearson colocalization coefficients in the lysosome and endoplasmic reticulum were 0.41 and 0.33, respectively, indicating that Vis-H_2_S exhibits good mitochondrial localization and could detect the response to viscosity and H_2_S in the mitochondria during cellular experiments.

We then investigated the ability of Vis-H_2_S (10 μM) to respond to exogenous and endogenous H_2_S and cell viscosity. The results are given in Fig. S13.† First cells were treated with sulfhydryl scavenger *N*-ethylmaleimide^20^ (NEM, 0.5 mM) and then incubated with different concentrations of H_2_S for 40 min. The experimental results indicated that upon increasing the NaHS (H_2_S donor) concentration (0, 5, 10, or 20 μM), *I*_green_ gradually decreased and *I*_red_ gradually increased, indicating that Vis-H_2_S could detect exogenous H_2_S in cells. Compared with the control group, the fluorescence intensity was significantly reduced for cells in the presence of NEM (0.5 mM). This was because of the removal of endogenous H_2_S by NEM. Then, in the absence of H_2_S due to the addition of NEM (reduced interference by H_2_S), intracellular viscosity levels were regulated using monensin and different cell incubation temperatures (25 °C and 4 °C; a lower temperature increases the intracellular viscosity). As shown in Fig. S14,† upon decreasing the temperature and adding monensin, *I*_green_ increased significantly, while *I*_red_ was almost negligible. These experiments confirm that Vis-H_2_S can readily respond to cell viscosity.

Next, we evaluated the dual-response of Vis-H_2_S to H_2_S and autophagy at the cellular level. Previous studies have shown that during autophagy, mitochondria are wrapped by vesicles to form autophagosomes that then fuse with lysosomes to form autolysosomes.^21^ This allows mitochondria and their contents to be degraded, resulting in reduced mitochondrial viscosity.^22^ As shown in Fig. S15,† compared with the control group and NEM group, after cells were treated with autophagy inducer rapamycin (100 nM, mTORC1 complex inhibitor^23^), *I*_green_ was negligible, indicating that autophagy significantly reduced the mitochondrial viscosity. In addition, different concentrations of NaHS (5, 10, or 20 μM) did not affect the *I*_green_, but *I*_red_ gradually increased with an increase in H_2_S concentration, indicating that Vis-H_2_S could generate a dual response to autophagy and H_2_S.

**Fig. 2:**
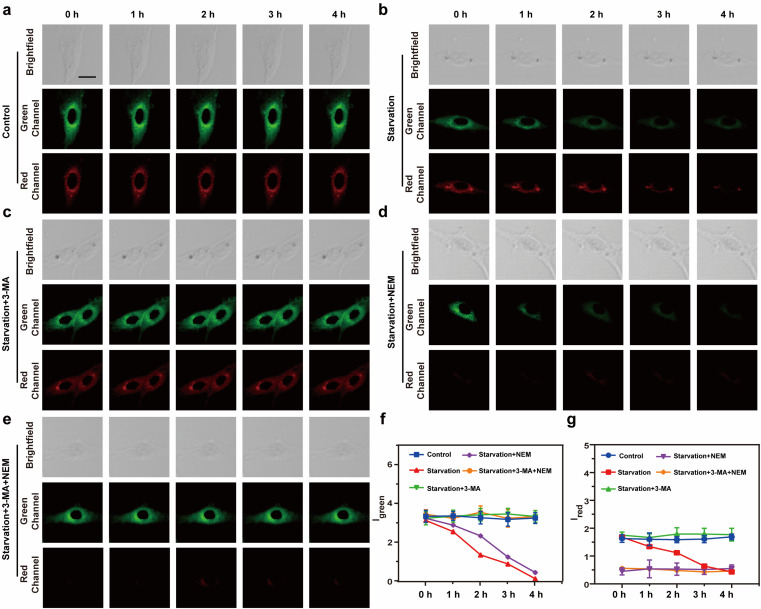
Real-time fluorescence imaging images of the HepG2 cells in: (a) control group, (b) starvation treatment, (c) starvation treatment + 5 mM 3-MA, (d) starvation treatment + 0.5 mM NEM, and (e) starvation treatment + 5 mM 3-MA + 0.5 mM NEM incubated with Vis-H_2_S (10 μM). (f) Fluorescence trend graph of intracellular *I*_green_ as a function of time. (g) Fluorescence trend graph of intracellular *I*_red_ as a function of time. Light collection range: green channel 450–520 nm, red channel 650–720 nm, the excitation light source is 552 nm, and the scale bar is 20 μm.

To further evaluate the ability of Vis-H_2_S to monitor autophagy and H_2_S in real-time, confocal real-time imaging analysis was performed on HepG2 cells stained with Vis-H_2_S at different times and under cell culture conditions. The results are shown in Fig. 2a–e. The fluorescence of the red and green channels of the control group (HepG2 cells were untreated) remained almost constant for HepG2 cells loaded with Vis-H_2_S with a 5 min interval for a total of 60 min, indicating that Vis-H_2_S possesses good photostability. The cells were then starved to induce autophagy,^24^ and the results indicated that both *I*_green_ and *I*_red_ were significantly reduced. From the above experimental results, a decrease in *I*_green_ occurred due to a decrease in mitochondrial viscosity during autophagy. In addition, the gradual decrease in *I*_red_ is attributed to the role of NFS1 protein as a reductive protein that facilitates the release of H_2_S through desulfuration of l-cysteine. During the autophagy process, lysosomes degrade NFS1 protein within the mitochondria, resulting in a reduction of endogenous H_2_S and subsequently weakening of *I*_red_. After the cells were starved and the autophagy inhibitor 3-methyladenine^25^ (3-MA, 5 mM) was added, both *I*_green_ and *I*_red_ were significantly enhanced and the fluorescence intensity remained almost unchanged for the next 60 min. This again shows that autophagy can simultaneously reduce mitochondrial viscosity and H_2_S levels. When cells were pre-incubated with NEM, regardless of whether the cells were incubated with 3-MA, changes in *I*_green_ were the same as those without NEM, while *I*_red_ was almost negligible. Due to the ability of flow cytometry to rapidly analyze a large number of individual cells, achieving high-throughput data collection is crucial for comprehensive assessment of fluorescence signal changes. In this regard, our study employed flow cytometry to investigate fluorescence intensity alterations under the aforementioned conditions, as illustrated in Fig. S16.† In comparison to the control group, cells subjected to starvation exhibited a significant decrease in fluorescence intensity in both the green and red channels, with the addition of 3-MA inhibiting this process. Concurrent starvation and NEM incubation resulted in a notable reduction in fluorescence intensity in both channels, with 3-MA incubation during this process leading to an increase in green channel fluorescence intensity and minimal interference with red channel fluorescence intensity. These findings align with real-time imaging results, confirming the dual-responsive capability of Vis-H_2_S to simultaneously monitor cellular viscosity and H_2_S levels.

**Fig. 3:**
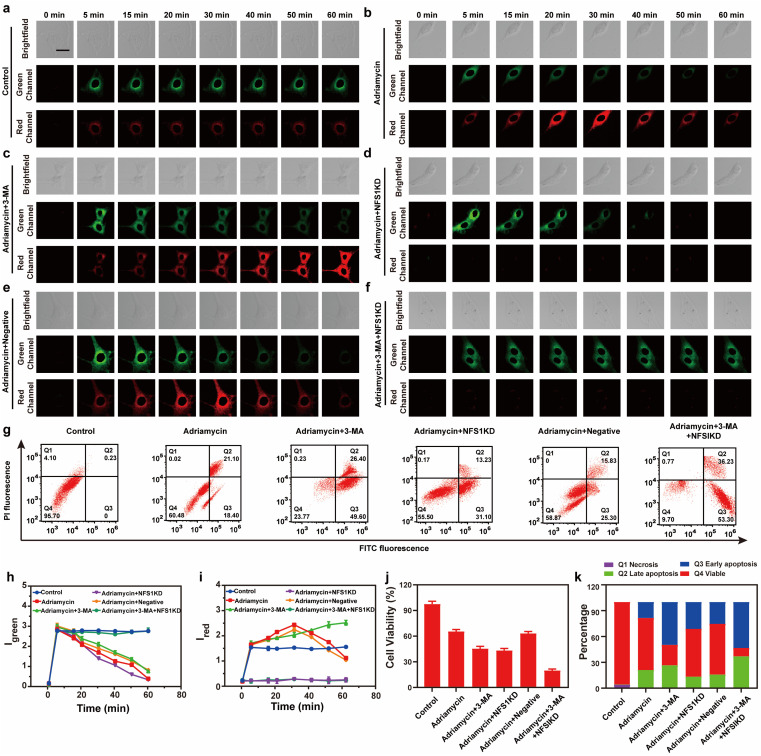
(a–f) Real-time imaging fluorescence image of the HepG2 cells in (a) control group, (b) Adriamycin (5 μM), (c) Adriamycin (5 μM) + 3-MA (5 mM), (d) Adriamycin (5 μM) after cysteine desulfurase (NFS1) gene knockout, (e) negative control of NFS1 and (f) Adriamycin (5 μM) + 3-MA (5 mM) after NFS1 gene knockout. (g) Apoptosis assay of cells in (a–f); viable cells (FITC^−^/PI^−^), early apoptosis (FITC^+^/PI^−^), late apoptosis (FITC^−^/PI^+^), and necrosis (FITC^+^/PI^+^). The time-dependence of the relative fluorescence intensity of (h) *I*_green_ and (i) *I*_red_. (j) MTT test of the HepG2 cells corresponding to (a–f). (k) Quantification data of the apoptosis assay results corresponding to (g).

Finally, we evaluated the source of MDR during tumor chemotherapy. Adriamycin, a widely used chemotherapeutic drug, works by consuming oxygen to produce ROS, which in turn triggers autophagy (ROS has been confirmed as the main cause of upregulating cellular autophagy) and overexpression of NFS1 protein due to increased hypoxia in tumor cells.^26^ This combination of events leads to MDR but the synergistic effect between cellular autophagy and NFS1 protein overexpression in causing MDR has not been previously reported. To investigate the combined impact of autophagy and NFS1 protein on multidrug resistance (MDR) during tumor chemotherapy, we employed Vis-H_2_S for imaging. Before conducting imaging experiments, we initially employed western blot analysis to assess the NFS1 protein levels. This step aimed to validate our hypothesis that the upregulation of NFS1 protein, induced by Adriamycin, leads to a substantial increase in H_2_S production. The mechanism suggests that elevated NFS1 levels act as a protective response in tumor cells, preventing the cytotoxic effects of Adriamycin and thereby contributing to the development of multidrug resistance. The results are depicted in Fig. S18,† where the addition of Adriamycin led to a significant increase in NFS1 protein content, while NFS1KD rendered the NFS1 protein content negligible. As shown in Fig. 3a–f, 3h and i, the control group experiment indicates that Vis-H_2_S retained excellent photostability (Fig. 3a). After adding Adriamycin, *I*_green_ was significantly reduced, while *I*_red_ increased first and then decreased (Fig. 3b). This occurred because the addition of Adriamycin significantly increased the overexpression of NFS1 protein. Then upon prolonged incubation time, autophagy decreased the mitochondrial viscosity resulting in decreased *I*_red_. To validate this result, cells incubated with Adriamycin were also treated with the autophagy inhibitor 3-MA (10 μM) or the NFS1 gene of HepG2 cells was eliminated (NFS1KD, operational approach according to the reported method^27^). The results indicated that *I*_green_ gradually decreased and *I*_red_ increased gradually in cells pretreated with 3-MA (Fig. 3c). This confirms that the fluorescence intensity was only regulated by NFS1-induced H_2_S. In the Adriamycin + NFS1KD group, *I*_green_ gradually decreased, and *I*_red_ was negligible, indicating that the fluorescence intensity was only regulated by autophagy. Compared with the Adriamycin group, the Adriamycin + NFS1KD group exhibited a more pronounced decrease in *I*_green_. We believe that the knockout of the NFS1 gene led to an increase in the cellular ROS level, which increased the autophagy level. In addition, the results of the negative control (performed according to the previous report^28^) group were similar to those of the Adriamycin group, indicating that the NFS1KD group results were caused by the reduction of H_2_S levels *via* the knockout of the NFS1 protein. Finally, when autophagy and NFS1 protein were simultaneously inhibited (as shown in Fig. 3f), unsurprisingly, *I*_green_ remained almost unchanged, while *I*_red_ was negligible. We then further validated the accuracy of the results using flow cytometric analysis, as illustrated in Fig. S17.† The addition of Adriamycin resulted in a significant decrease in fluorescence intensity in both the green and red channels. Under these conditions, inhibiting autophagy (3-MA) led to a significant increase in red channel fluorescence intensity, while inhibiting NFS1 protein levels (NFS1KD) resulted in a significant decrease in red channel fluorescence intensity. This aligns with the real-time imaging results, demonstrating the probe's capability to detect cellular autophagy and NFS1 level fluctuations during the development of Adriamycin-induced drug resistance.

Having ascertained the autophagy and NFS1 protein levels under the given conditions, this study also employed Annexin V-FITC/PI apoptosis detection agent assay and the CCK8 assay to comprehensively evaluate the Adriamycin drug resistance under different conditions. As shown in Fig. 3g, 3j and k, the cells in the control group maintained a high survival rate, while the survival rate of the cells in the Adriamycin group decreased to 60.5%. Interestingly, when autophagy was inhibited or when the NFS1 protein was knocked out, the apoptosis rate was significantly increased to 49.6% and 31.1%, respectively. In addition, the lowest cell survival rate (9.7%) was achieved by the simultaneous inhibition of autophagy and knockdown of the NFS1 protein. The above experiments indicate that autophagy and the NFS1 protein are responsible for Adriamycin drug resistance.

## Conclusions

A near-infrared fluorescence-based probe, Vis-H_2_S, which could simultaneously respond to H_2_S and viscosity, was constructed based on the QCy7 parent fluorophore. The probe could simultaneously monitor autophagy and H_2_S during the treatment of cells with Adriamycin and further confirmed the direct relationship between autophagy and NFS1 protein overexpression with ROS-based chemotherapeutic drugs. The results indicated that Adriamycin up-regulated the degree of autophagy and the NFS1 protein levels during tumour treatment, which resulted in MDR. Furthermore, reducing the level of NFS1 protein results in a small increase in autophagy levels, which may be because the NFS1 protein is unable to fully clear the ROS. Hence, using probe Vis-H_2_S we could confirm that both autophagy and NFS1 protein are closely related to the MDR of tumour cells. Therefore, Vis-H_2_S is a powerful analytical tool for the in-depth exploration of the drug resistance mechanism of Adriamycin.

## Data availability

All data supporting this study are provided as ESI† accompanying this paper.

## Author contributions

Wei Hu: conceptualization, investigation, methodology, data curation, writing – original draft. Yifan He: conceptualization, data curation. Haixian Ren: conceptualization, supervision, funding acquisition. Li Chai: validation, data curation. Haiyan Li: methodology, data curation. Jianbin Chen: validation, funding acquisition. Chunya Li: supervision, validation, funding acquisition. Yanying Wang: supervision, validation. Tony D. James: conceptualization, validation, supervision, project administration, writing – review and editing.

## Conflicts of interest

There are no conflicts to declare.

## Supplementary Material

SC-015-D3SC06459J-s001
