# Supplementary material for: Near-infrared imaging for visualizing the synergistic relationship between autophagy and NFS1 protein during multidrug resistance using an ICT–TICT integrated platform
Source: Chem Sci. 2024 Mar 25;15(16):6028–35. doi: 10.1039/d3sc06459j (PMC11040642; doi:10.1039/d3sc06459j)
Supplement: SC-015-D3SC06459J-s001 [file SC-015-D3SC06459J-s001.pdf]

Electronic Supplementary Material (ESI) for Chemical Science.
This journal is © The Royal Society of Chemistry 2024

Near-infrared imaging for visualizing the synergistic relationship between autophagy and NFS1 protein during multidrug resistance using an ICT-TICT integrated platform

Wei Hu [a, b, c, //], Yifan He [a, //], Haixian Ren [b, *], Li Chai [b], Haiyan Li [a], Jianbin Chen [d], Chunya Li [a, *], Yanying Wang [a, *], Tony D. James [c, e, *]

[a.] Key Laboratory of Catalysis and Energy Materials Chemistry of Ministry of Education & Key Laboratory of Analytical Chemistry of the State Ethnic Affairs Commission, College of Chemistry and Materials Science, South-Central Minzu University, Wuhan 430074, China.

[b.] Department of Chemistry, Xinzhou Normal University, Xinzhou, Shanxi, 034000, China.

[c.] Department of Chemistry, University of Bath, Bath, BA27AY, United Kingdom.

[d.] school of Chemistry and Chemical Engineering, Qilu University of Technology (Shandong Academy of Sciences), Jinan, Shandong 250353 China.

[e]. School of Chemistry and Chemical Engineering, Henan Normal University, Xinxiang 453007, China,

*Corresponding E-mail address: hxren326@163.com. (H. X. Ren); lichychem@mail.scuec.edu.cn. (C. Y. Li); wangyychem@mail.scuec.edu.cn (Y. Y. Wang) and t.d.james@bath.ac.uk. (T. D. James).

‡ These authors contributed equally to this paper.

Table of Contents

1. General Information on Materials and Methods 3

Instruments and materials 3

Spectroscopic measurements 3

Viscosity determination and fluorescence measurements.......................................4

Determination of the detection limit 4

Cell culture and imaging 4

Flow Cytometric Analysis..4

2. Synthesis of Vis-H_2S..5

3. Supplementary Figures...6

4. Reference...19

1. General Information on Materials and Methods

Instruments and materials

Unless otherwise stated, all solvents and reagents were purchased from commercial suppliers and were used as received without further purification. HepG2 cells were obtained from Procell Life Science & Technology Co., Ltd. 3-(4,5-Dimethylthiazol-2-yl)-2,5-diphenyltetrazolium bromide (MTT), Green commercial co-localization dyes (Lyso-Tracker Red for lysosomes, Mito-Tracker Red for mitochondria, and ER-Tracker Red for the endoplasmic reticulum), Rapamycin, N-Ethylmaleimide, 3-Methyladenine, Monenmin, NaSH, Hank's balanced salt solution (HBSS), Malonic, Adriamycin, 2-Acetoxy-1-methoxypropane were purchased from Sigma-Aldrich. All aqueous solutions were prepared by ultrapure water with a resistivity of 18.25 MΩ cm (purified by Milli-Q system, Millipore). High-resolution mass spectrometry was performed with LTQ FT Ultra (Thermo Fisher Scientific, America) in MALDI-DHB mode. NMR spectra were recorded on a Bruker-400 spectrometer, using TMS as an internal standard. Absorption spectra were recorded with a UV-vis spectrophotometer (Shimadzu UV-2550, Japan), and one-photon fluorescence spectra were obtained with a fluorimeter (Shimadzu RF-6000, Japan). One photon microscopy was performed on a Zeiss LSM 710 multiphoton laser scanning confocal microscope (Carl Zeiss, Germany).

Spectroscopic measurements

Unless otherwise mentioned, all the measurements using **Vis-H_2S** target reaction were tested in PBS buffer (10 mM, pH 7.4, containing 0.9% NaCl)/glycerol system. The excitation wavelengths were 400 nm under one-photon excitation mode, respectively.

The $ONOO^-$ source was the donor 3-morpholinosydnonimine hydrochloride (SIN-1, 200 mM)[1]. NO was generated in form of 3-(aminopropyl)-1-hydroxy-3-isopropyl-2-oxo-1-triazene (NOC-5, 100 μM)[2]. H_2O_2 was determined at 240 nm ($\varepsilon_{240\ nm}$ = 43.6 $M^{-1}cm^{-1}$)[3]. NO_2 was generated from $NaNO_2$[4]. All the reagents were obtained from Aladdin (USA). All other chemicals were from commercial sources and of analytical reagent grade unless indicated otherwise.

Viscosity determination and fluorescence measurements

The solvents were obtained by mixing an PBS-glycerol system in different proportions. Measurements were carried out with an NDJ-8S rotational viscometer, and each viscosity value was recorded[5]. The solutions of **Vis-H_2S** of different viscosity were prepared by adding the stock solution (1.0 mM) 10 µL to 1 mL of solvent mixture (PBS-glycerol solvent systems) to obtain the final concentration of **Vis-H_2S** (10.0 µM). These solutions were sonicated for 5 minutes to eliminate air bubbles. After standing for 1 hour at a constant temperature, the solutions were measured in a UV spectrophotometer and a fluorescence spectrophotometer.

Determination of the detection limit

The limit of detection (LOD) for H_2O_2 was calculated based on the following equation[6]:

$$LOD = 3\sigma/k$$

Where σ represents the standard deviation of the black (n = 11) and k represents the slope of the titration spectra curve among the limited range.

Cell culture and imaging

HepG2 cells were cultured in Dulbecco's modified Eagle's medium (DMEM, Thermo Scientific) supplemented with 1% penicillin/streptomycin and 10% fetal bovine serum (FBS), and incubated in an atmosphere of 5/95 (v/v) of CO_2/air at 37 °C. Two days before imaging, the cells were passed and placed into glass-bottomed dishes (NEST). For labeling, the cells were washed with serum-free DMEM and then incubated with 10 µM **Vis-H_2S** (containing 1% DMSO) for 30 min at 37 °C.

Flow Cytometric Analysis

The HepG2 cells were cultured at 1.0×10^6 cells/well in 6-well plates, and then treated with different concentration of $NaHSO_3$ as described in the paper. After harvesting, cells were washed and suspended in fresh complete medium. 5 µL AnnexinV-FITC was mixed, then add 5 µL PI mix and analyzed by flow cytometry.

2. Synthesis of Vis-H_2S

Compound **1** was synthetized according to our previously reported method.[7]

Synthesis of compound **2**. 2,4-diformylphenyl acetate (384.1 mg, 2 mmol), sodium acetate (541.6 mg, 3.98 mmol), 693.4 mg (3.98 mmol) of compound 1 and 10 mL of acetic anhydride in a 50 mL round bottom flask, heated at 80 ^{o}C for 30 min under nitrogen. After the reaction, the solvent was evaporated under reduced pressure. The residue was washed with distilled water, dried, and separated by silica gel column chromatography with dichloromethane/methanol (10/1, V/V) as eluent. 273.6 mg of compound **2** was obtained (679.2 mg, yield: 45%). ^{1}H NMR (400 MHz, DMSO-d_6) δ 7.94 (dd, J = 14.6, 2.6 Hz, 2H), 7.73 (d, J = 1.9 Hz, 1H), 7.70 (d, J = 14.7 Hz, 1H), 7.67 – 7.62 (m, 3H), 7.61 – 7.56 (m, 3H), 7.53 – 7.48 (m, 4H), 7.19 (d, J = 8.6 Hz, 1H), 4.23 (s, 6H), 2.31 (s, 3H), 1.70 (s, 12H).

Synthesis of **Vis-H_2S**. O-Iodobenzoic acid (16.3 mg, 0.066 mmol) was dissolved in dichloromethane, *N*, *N*-dicyclohexylcarbodiimide (16.3 mg, 0.079 mmol) and 4-dimethylaminopyridine (0.81 mg, 0.006 mmol) were added in an ice bath. and after stirring for 30 minutes, compound 2 (50 mg, 0.066 mmol) was added, and the mixture was stirred at room temperature overnight. The reaction progress was determined by TLC. After the reaction was complete, the product was purified by silica gel column chromatography (40.6 mg, yield: 65%).^{1}H NMR (400 MHz, DMSO-d_6) δ 8.02 (dd, J = 7.5, 1.6 Hz, 1H), 7.94 (dd, J = 14.6, 2.8 Hz, 2H), 7.80 (dd, J = 7.5, 1.5 Hz, 1H), 7.76 (d, J = 1.9 Hz, 1H), 7.67 – 7.56 (m, 6H), 7.54 – 7.44 (m, 6H), 7.37 (td, J = 7.4, 1.5 Hz, 1H), 7.24 (td, J = 7.4, 1.5 Hz, 1H), 4.23 (s, 6H), 1.70 (s, 12H). ^{13}C NMR (100 MHz, DMSO-d_6) δ 167.74, 164.79, 164.27, 150.01, 148.17, 145.92, 145.89, 141.05, 141.00, 140.23, 132.08, 131.82, 131.28, 131.19, 131.16, 129.69, 128.57, 128.33, 128.27, 128.20, 127.26, 126.81, 126.70, 125.16, 125.14, 121.17, 120.62, 119.23, 117.51, 117.50, 98.46, 54.46, 54.44, 36.52, 36.50, 27.11, 27.09, 27.07. $[M]^{2+}$ calcd for $[C_{39}H_{37}N_2O_2I]$ 346.0944, found 346.1209.

3. Supplementary Figures

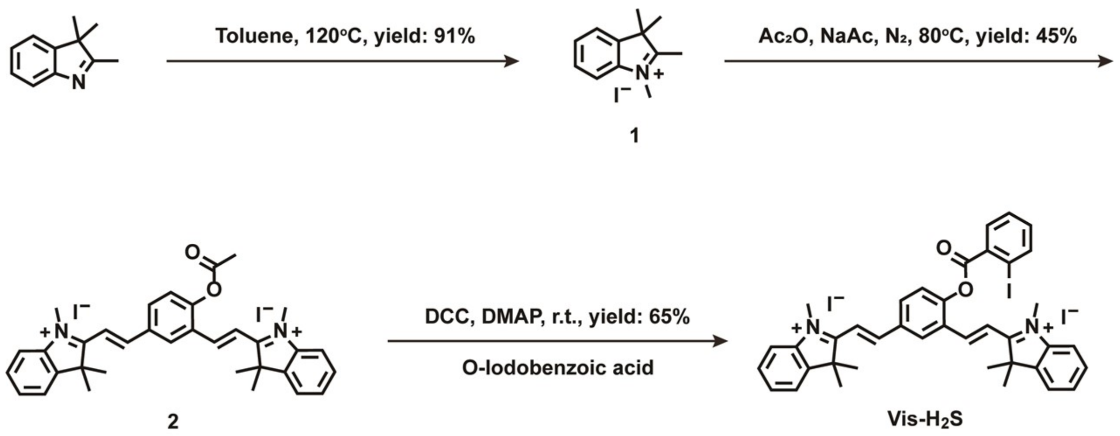

Scheme S1. Synthetic procedure for **Vis-H_2S**.

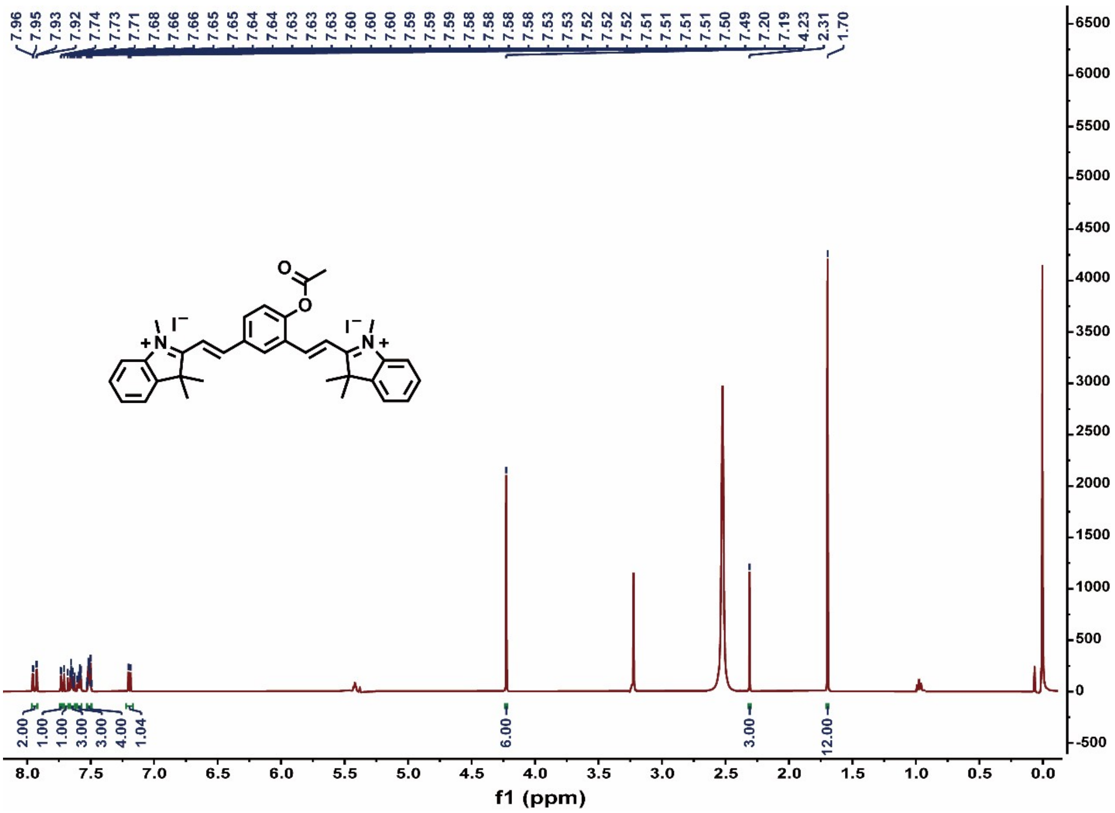

Figure S1. ^{1}H NMR spectrum of Compound **2** (DMSO-d_6).

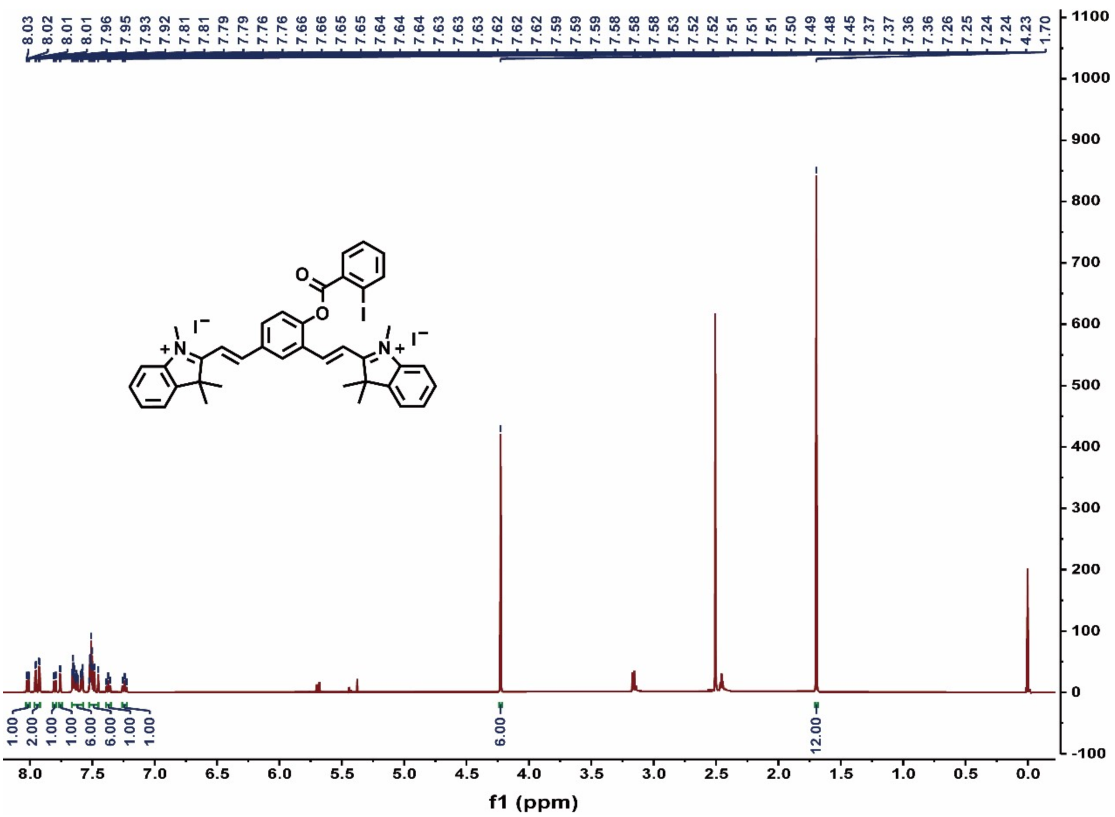

Figure S2. ^{1}H NMR spectrum of **Vis-H$_2$S** (DMSO-d_6).

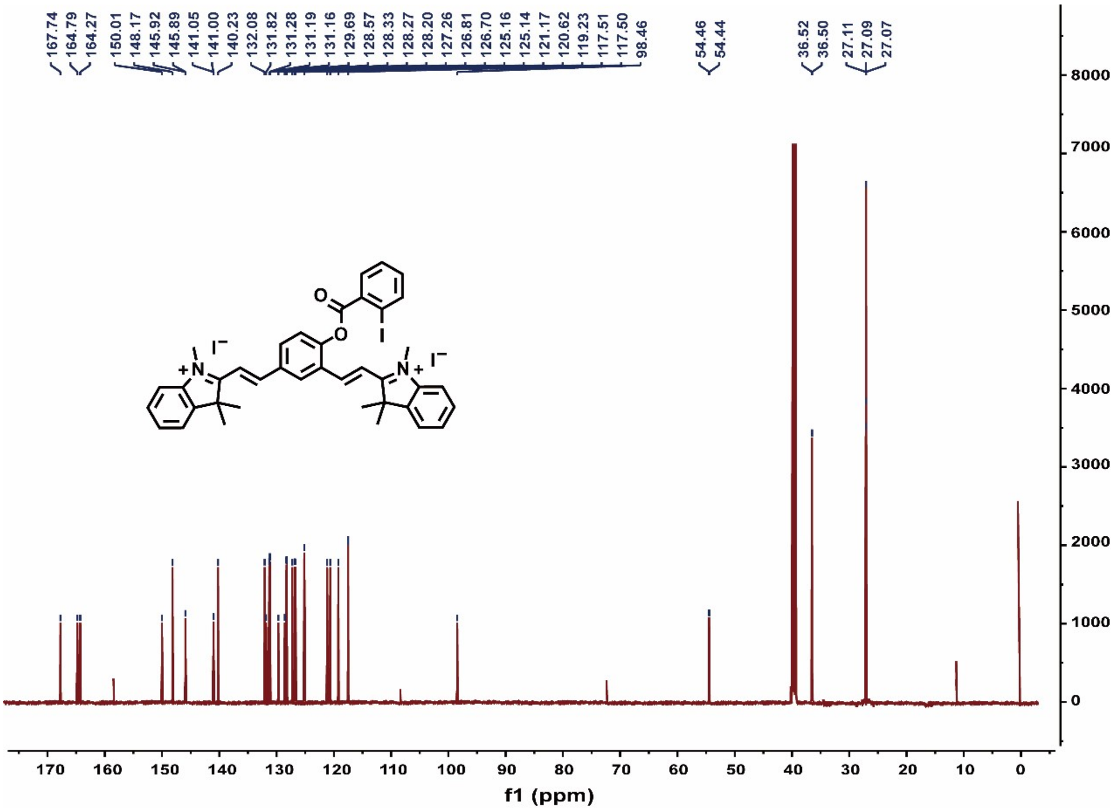

Figure S3. ^{13}C NMR spectrum of **Vis-H$_2$S** (DMSO-d_6).

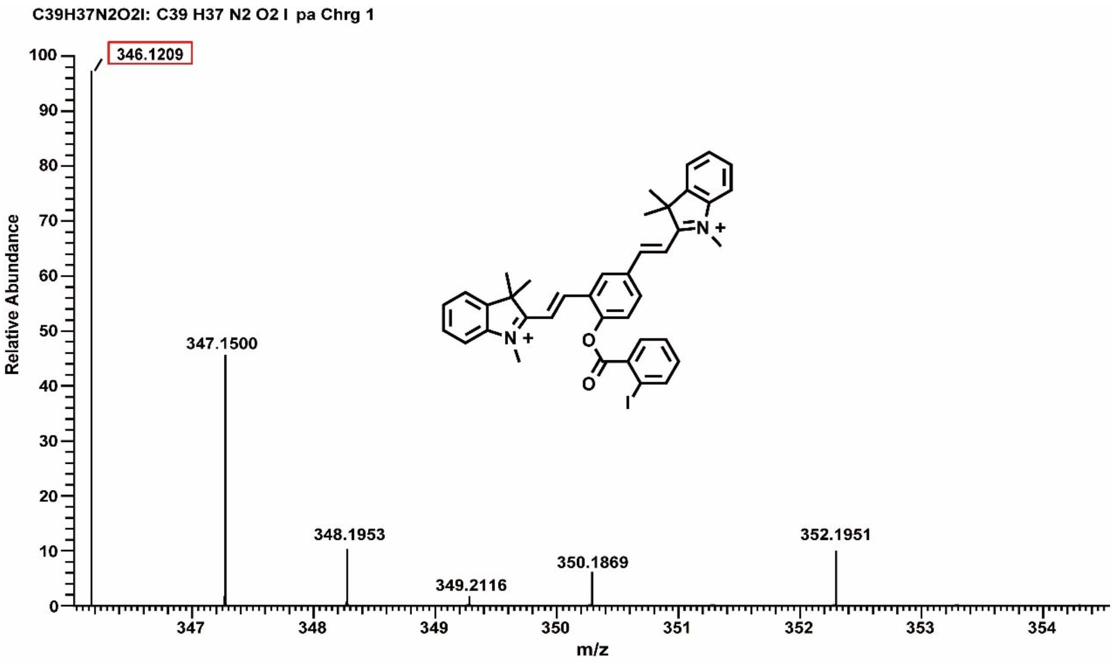

Figure S4. HRMS spectrum of **Vis-H_2S**.

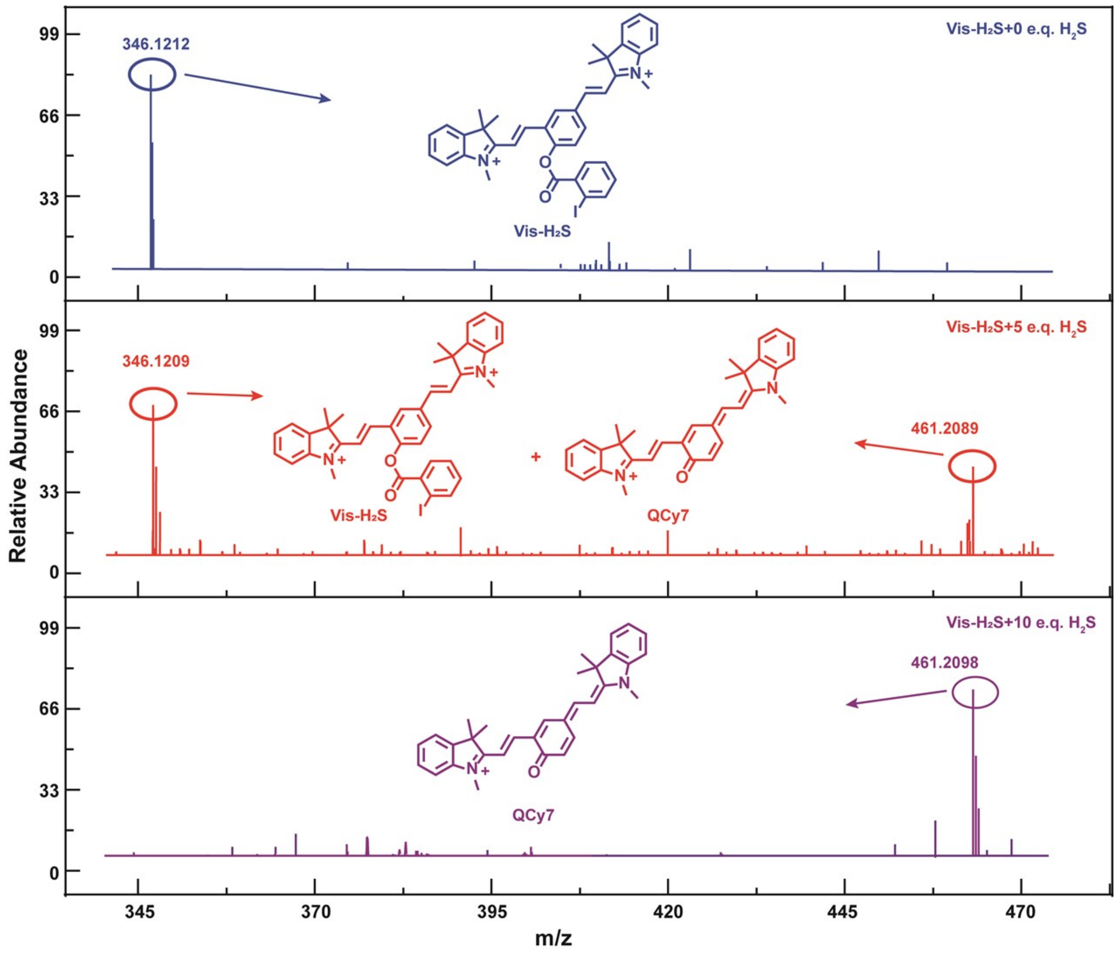

Figure S5. HRMS spectrum of **Vis-H_2S** with H_2S.

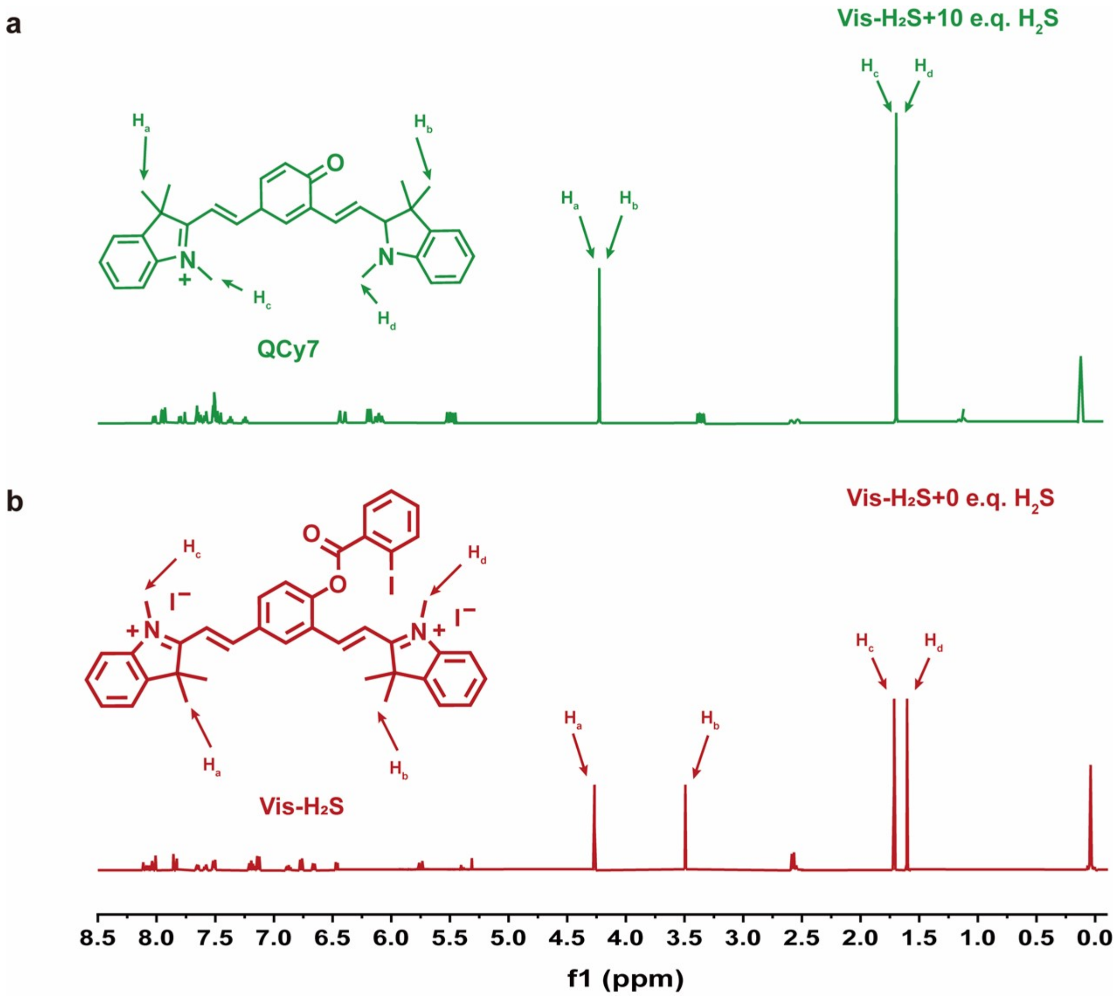

Figure S6. ^{1}H NMR spectrum of **Vis-H$_2$S** with H_2S.

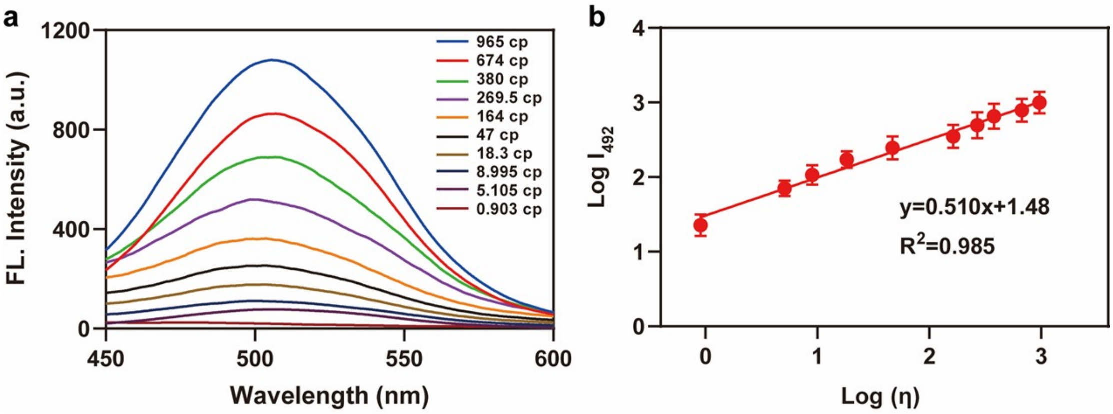

Figure S7. a) Fluorescence spectra of **Vis-H_2S** viscosity response; b) Linear relationship between the relative fluorescence intensity of the probe and the viscosity; Excitation wavelength: 400 nm; Emission wavelength: 450 nm-600 nm.

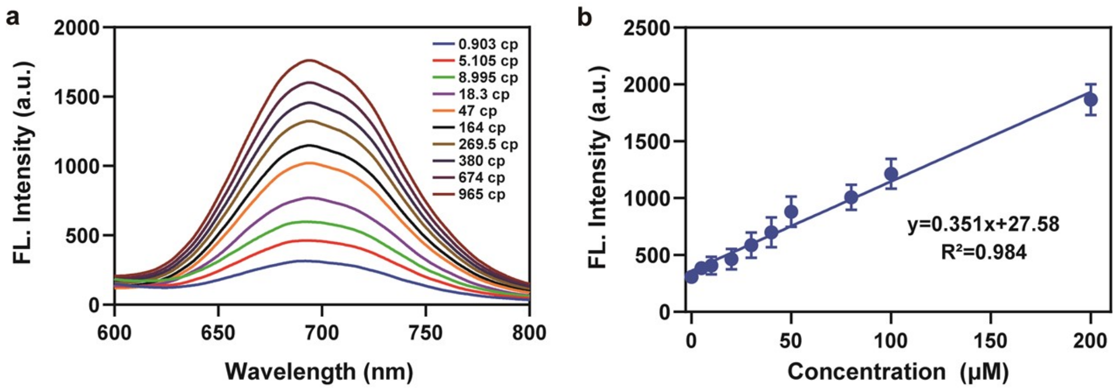

Figure S8. a) Fluorescence spectra of QCy7 viscosity response; b) Linear relationship between the relative fluorescence intensity of the probe and the viscosity; Excitation wavelength: 400 nm; Emission wavelength: 600 nm-800 nm.

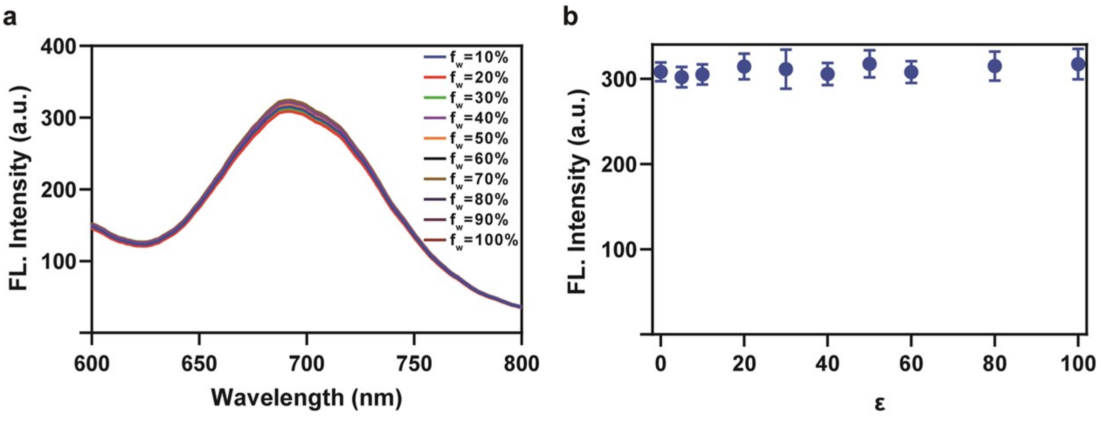

Figure S9. a) Fluorescence spectra of **QCy7** in THF/PBS mixtures with different volume fractions (f_w) of PBS; b) Plot of the fluorescence intensity of **QCy7** (λ_{em} = 687 nm) versus the dielectric constant (ε) of the solvent; Excitation wavelength: 400 nm; Emission wavelength: 600 nm-800 nm.

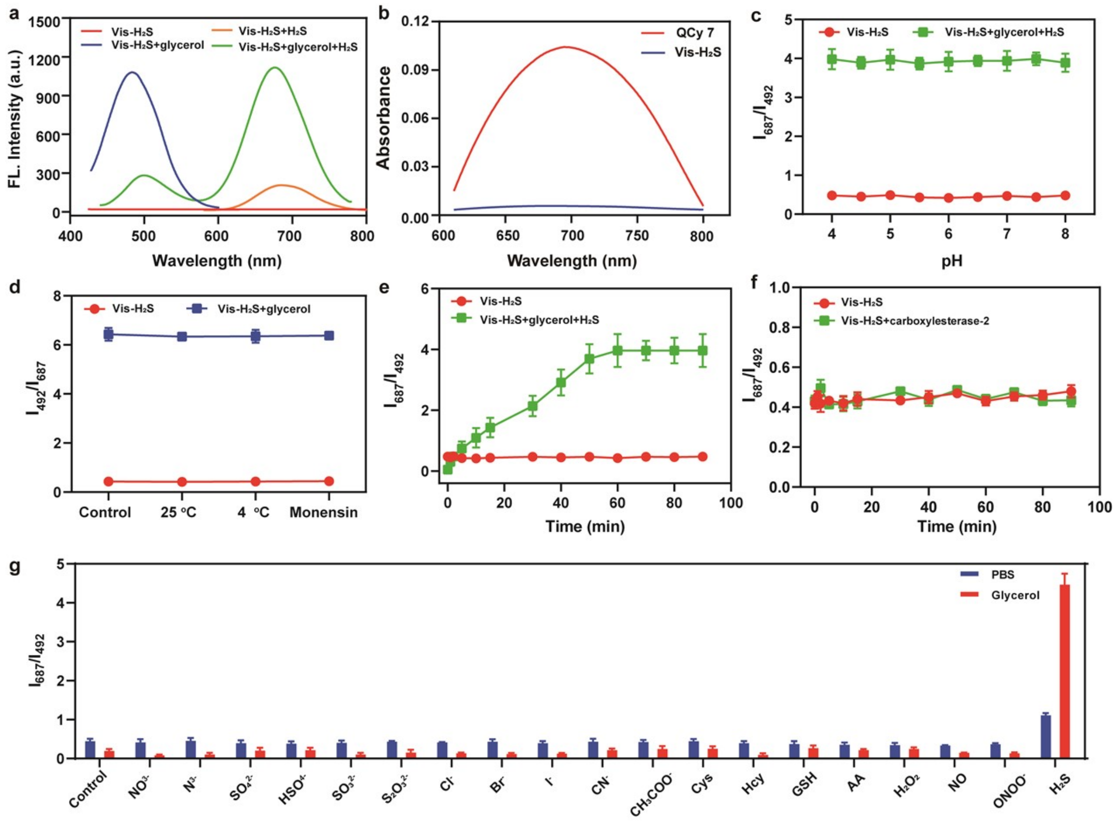

Figure S10. a) Fluorescence spectra of **Vis-H_2S** (10 μM) in response to H_2S and viscosity; b) Absorption spectra of **Vis-H_2S** (10 μM) and **QCy7** (10 μM); c) Under different pH conditions, the fluorescence intensity changes of **Vis- H_2S** before and after the reaction with H_2S (10 μM) and H_2S (200 μM)+70% glycerol; d) viscosity stability test after changing the aqueous solution environment; e) Time response of **Vis- H_2S** to H_2S (10 μM) and H_2S (200 μM)+70% glycerol; f) Photostability of the **Vis- H_2S** in the presence of carboxylesterase-2 (0.50 μg/mL); g) fluorescence histogram of the reaction of **Vis-H_2S** with different analytes.

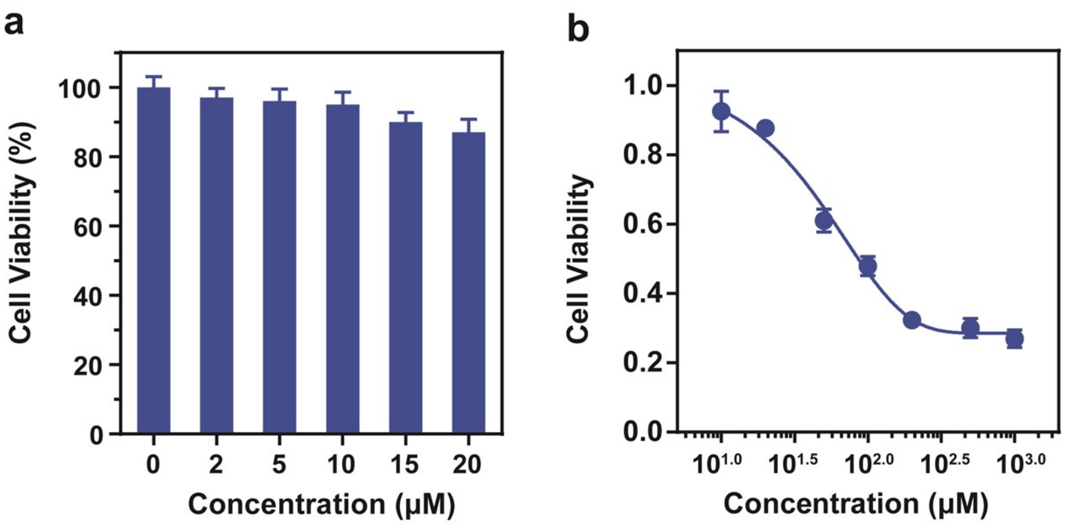

Figure S11. a) Viability of HepG2 cells was assessed by MTT assay following exposure to various concentrations of Vis-H_2S (0, 2, 5, 10, 15, 20 μM). Results are presented as the mean of triplicate measurements with error bars indicating standard deviation (SD). b) Inhibitory Concentration (IC_{50}) Determination for Vis-H_2S in HepG2 Cells. The inhibitory concentration (IC_{50}) of Vis-H_2S was determined over a concentration range of 10-1000 μM in HepG2 cells. The IC_{50} value, representing the concentration at which Vis-H_2S inhibits 50% of cell viability, was calculated. Each data point is the mean of three replicates, and error bars indicate the standard deviation (SD).

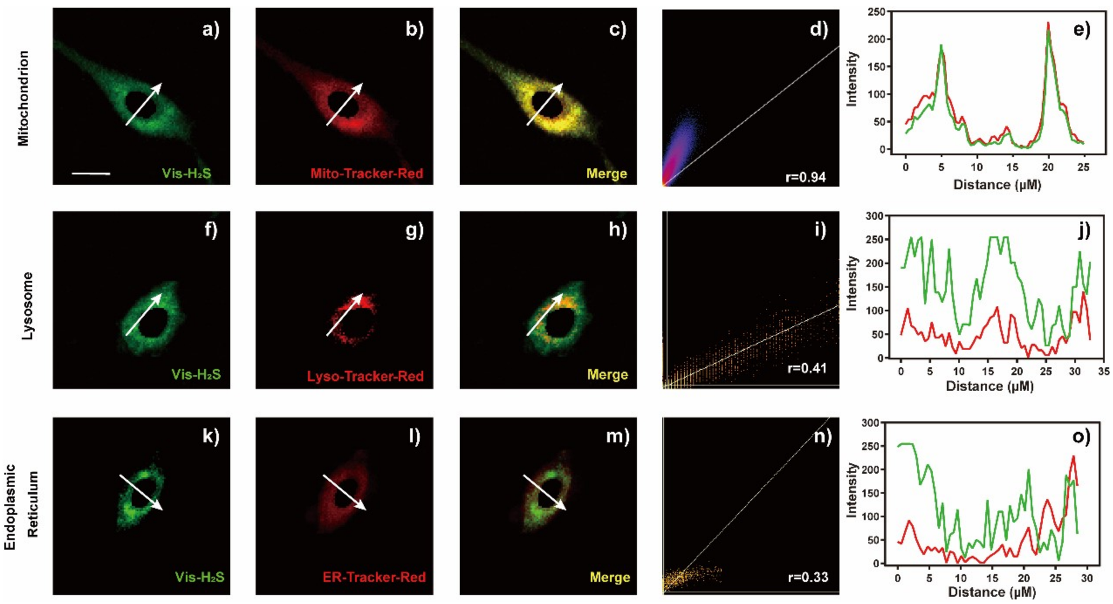

Figure S12. Confocal microscopy images of cells with **Vis-H$_2$S** (5 μM) and (a–c) Mito-Tracker Red, (f–h) Lyso-Tracker Red, and (k–m) ER-Tracker Red. (d, i, n) Fluorescence intensity correlation plot of **Vis-H$_2$S** and commercial dyes. Scale bar: 50 μm. (e, j, o) Fluorescence intensity profile of the region of interest across cells in the red and green channels.

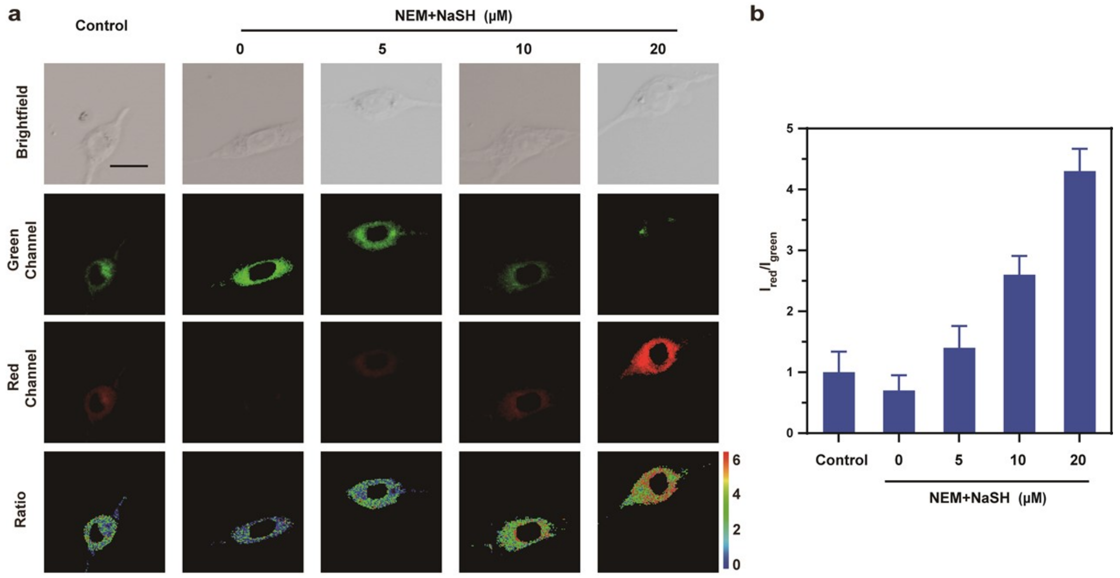

Figure S13. a) Confocal imaging of **Vis-H_2S** response to H_2S. In the control group, HepG2 cells were treated with **Vis-H_2S** (10 μM); in the other groups, HepG2 cells were first treated with NEM (0.5 mM) and then with different concentrations (0, 5, 10, 20 μM) of NaSH, and then the probe added. Confocal images of **Vis-H_2S** (10 μM) incubation; b) Ratio fluorescence intensity (ratio=I_{red}/I_{green}). Light collection range: green channel 450-520 nm, red channel 650-720 nm, excitation light source is 552 nm, scale bar is 20 μm.

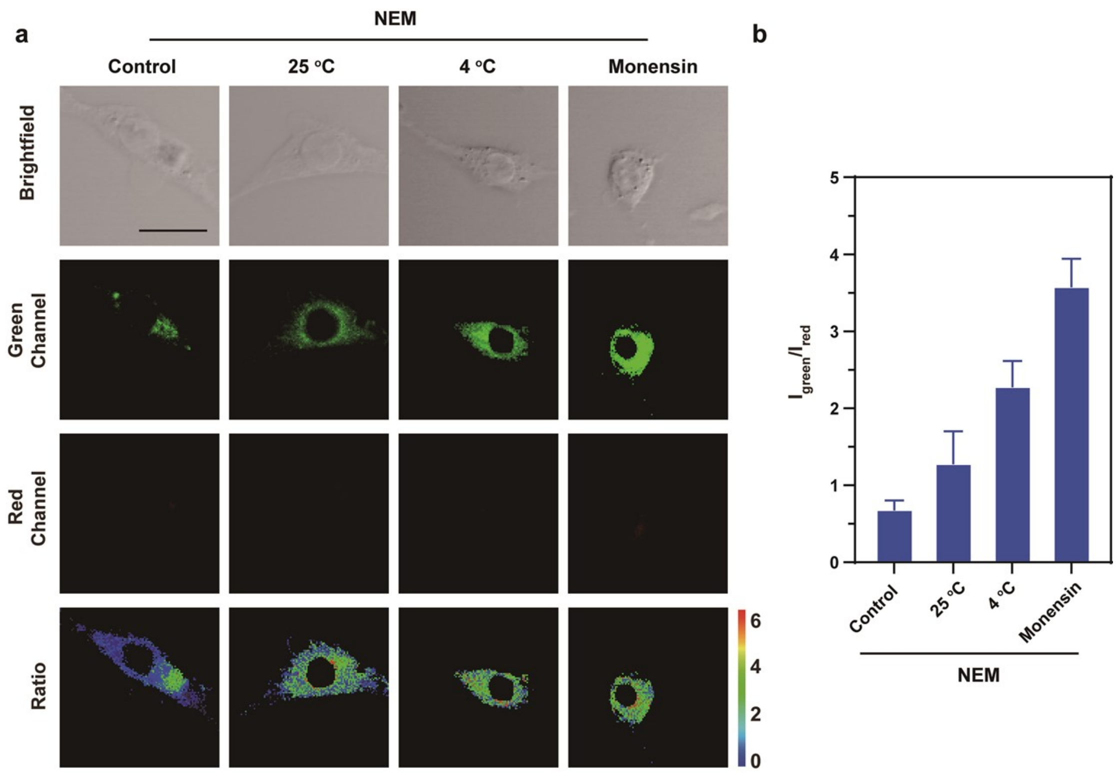

Figure S14. a) Confocal imaging of the viscosity response of **Vis-H_2S**. The image of the control group HepG2 cells treated with **Vis-H_2S** (10 µM) + NEM (0.5 mM); In the other groups, HepG2 cells were first treated with 4 °C + NEM (0.5 mM), 25 °C + NEM (0.5 mM), and monensin (10 µM) + NEM (0.5 mM), respectively, and then treated with probes. Confocal images of **Vis-H_2S** (10 µM) incubation; b) Ratio fluorescence intensity (ratio = I_{green}/I_{red}). Light collection range: green channel 450-520 nm, red channel 650-720 nm, excitation light source is 552 nm, scale bar is 20 µm.

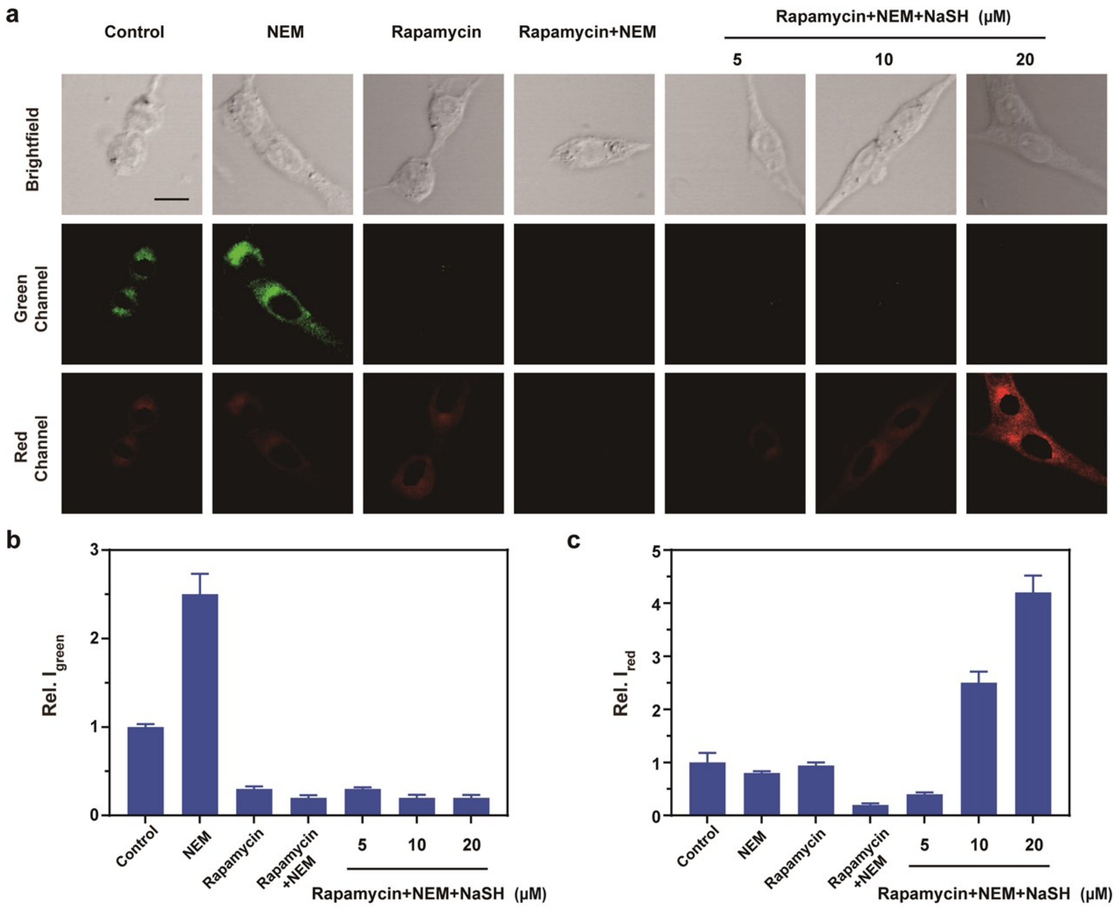

Figure S15. a) Confocal imaging of **Vis-H_2S** response to H_2S and viscosity. The image of the control group HepG2 cells treated with **Vis-H_2S** (10 μM); The other groups were treated with NEM (0.5 mM), Rapamycin (5 μM), NEM (0.5 mM) + Rapamycin (5 μM), NEM (0.5 mM) + Rapamycin (5 μM) + NaSH (5, 10, 20 μM) treatment followed by incubation with **Vis-H_2S** (10 μM). b) Histogram of relative fluorescence intensity of green channel. c) Histogram of relative fluorescence intensity of red channel. Light collection range: green channel 450-520 nm, red channel 650-720 nm, excitation light source is 552 nm, scale bar is 20 μm

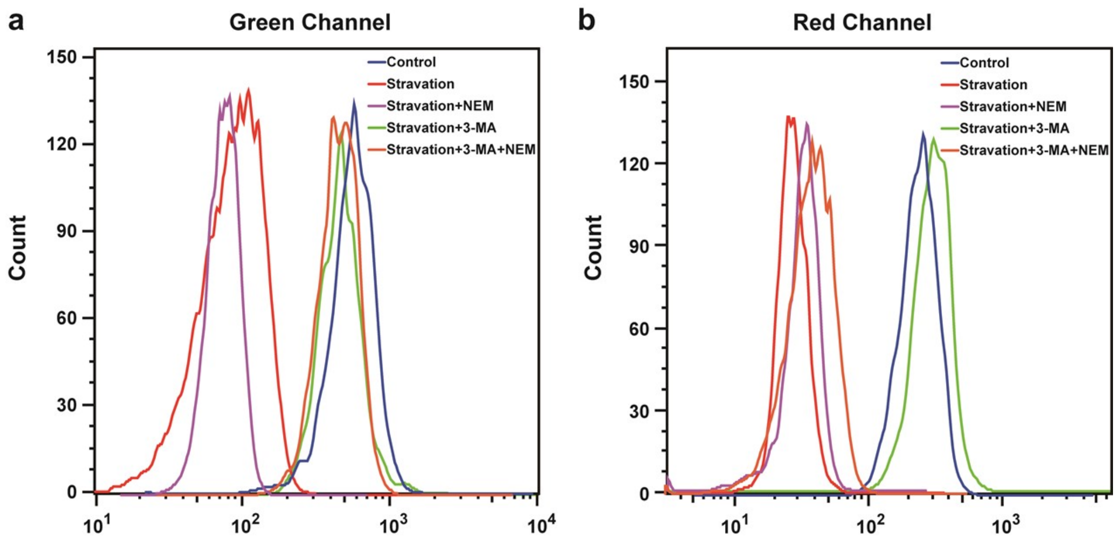

Figure S16. Flow cytometric analysis of fluorescence intensity in the green channel a) and red channel b). The cells used for this analysis are from the same source as depicted in Figure 2.

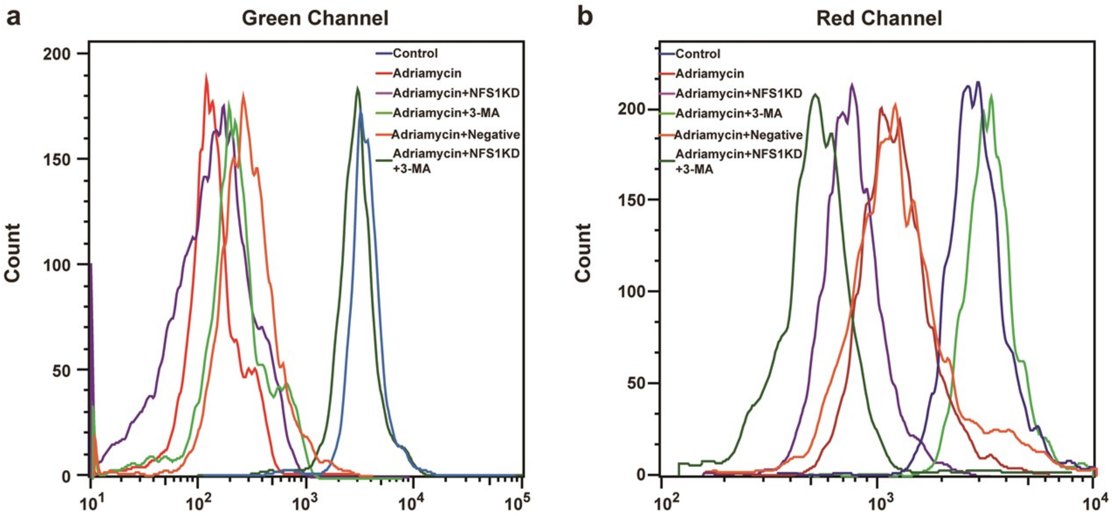

Figure S17. Flow cytometric analysis of fluorescence intensity in the green channel a) and red channel b). The cells used for this analysis are from the same source as depicted in Figure 3.

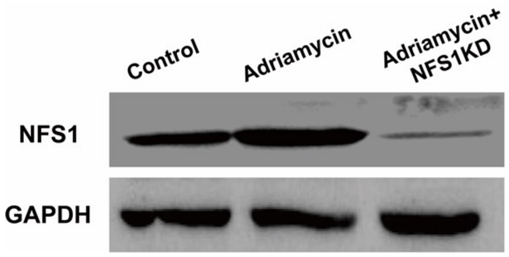

Figure S18. Western blotting illustrating the expression of cysteine desulfurase (NFS1) of the HepG2 cells in Control group, Adriamycin group and Adriamycin (5 μM) after NFS1 knockout (Adriamycin+ NFS1KD) group.

4. Reference

1 R. Q. Li, Z. Q. Mao, L. Rong, N. Wu, Q. Lei, J. Y. Zhu, L. Zhuang, X. Z. Hang and Z. H. Liu, *Biosens. Bioelectron.,* 2017, **87**, 73-80.

2 L. Y. Zeng, T. Xia, W. Hu, S. Y. Chen, S. Y. Chi, Y. D. Lei and Z. H. Liu, *Anal. Chem.,* 2018, **90**, 1317-1324.

3 X. T. Jia, Q. Q. Chen, Y. F. Yang, Y. Tang, R. Wang, Y. F. Xu, W. P. Zhu and X. H. Qian, *J. Am. Chem. Soc.,* 2016, **138**, 10778-10781.

4 Z. Q. Mao, W. Q. Feng, Z. Li, L. Y. Zeng, W. J. Lv and Z. H. Liu, *Chem. Sci.,* 2016, **7**, 5230-5235.

5 F. Cheng, T. T. Qiang, L. F. Ren, T. Y. Liang, X. Y. Gao, B. S. Wang and W. Hu, *Analyst.,* 2021, **146**, 2632-2637.

6 M. T. Ye, W. Hu, M. He, C. C. Li, S. Y. Zhai, Z. H. Liu, Y. Y. Wang, H. J. Zhang and C. Y. Li, *Chem. Commun.,* 2020, **56**, 6233-6236.

7 Y. F. He, W. Hu, L. Chai, Y. Y. Wang, X. Wang, T. Liang, H. Y. Li and C. Y. Li, *Chem. Commun.,* 2022, **58**, 7301-7304.
